# Expression of single-chain variable fragments fused with the Fc-region of rabbit IgG in *Leishmania tarentolae*

**DOI:** 10.1186/1475-2859-13-9

**Published:** 2014-01-15

**Authors:** Mathias Lindh Jørgensen, Niels Anton Friis, Jesper Just, Peder Madsen, Steen Vang Petersen, Peter Kristensen

**Affiliations:** 1 Department of Engineering, Aarhus University, Gustav Wieds Vej 10, Aarhus, Denmark; 2 Department of Molecular Biology and Genetics, Aarhus University, Gustav Wieds Vej 10, Aarhus, Denmark; 3 Department of Biomedicine, Aarhus University, Ole Worms Allé 3, Aarhus, Denmark

**Keywords:** Recombinant antibodies, Leishmania tarentolae, Protein expression

## Abstract

**Background:**

In recent years the generation of antibodies by recombinant methods, such as phage display technology, has increased the speed by which antibodies can be obtained. However, in some cases when recombinant antibodies have to be validated, expression in *E. coli* can be problematic. This primarily occurs when codon usage or protein folding of specific antibody fragments is incompatible with the *E. coli* translation and folding machinery, for instance when recombinant antibody formats that include the Fc-region are needed. In such cases other expression systems can be used, including the protozoan parasite *Leishmania tarentolae (L. tarentolae).* This novel host for recombinant protein expression has recently shown promising properties for the expression of single-chain antibody fragments. We have utilised the *L. tarentolae* T7-TR system to achieve expression and secretion of two scFvs fused to the Fc-region of rabbit immunoglobulin G (IgG).

**Results:**

Based on the commercial vector pLEXSY_IE-blecherry4 (Jena Bioscience; Cat. No. EGE-255), we generated a vector containing the Fragment Crystallisable (Fc) region of rabbit IgG allowing insertions of single chain antibody fragments (scFvs) in frame via *Nco*l/*Not*l cloning (pMJ_LEXSY-rFc). For the expression of rabbit Fc-fusion scFvs (scFv-rFc) we cloned two scFvs binding to human vimentin (LOB7 scFv) and murine laminin (A10 scFv) respectively, into the modified vector. The LOB7-rFc and A10-rFc fusions expressed at levels up to 2.95 mg/L in *L. tarentolae* T7-TR. Both scFv-rFcs were purified from the culture supernatants using protein A affinity chromatography. Additionally, we expressed three different scFvs without the rFc regions using a similar expression cassette, obtaining yields up to 1.00 mg/L.

**Conclusions:**

To our knowledge, this is the first time that antibody fragments with intact Fc-region of immunoglobulin have been produced in *L. tarentolae.* Using the plasmid pMJ_LEXSY-rFc, *L. tarentolae* T7-TR can be applied as an efficient tool for expression of rFc fusion antibody fragments, allowing easy purification from the growth medium. This system provides an alternative in cases where antibody constructs express poorly in standard prokaryotic systems. Furthermore, in cases where bivalent Fc-fused antibody constructs are needed, using *L. tarentolae* for expression provides an efficient alternative to mammalian expression.

## Background

Antibodies are applied in both basic research and diagnostics, and represent an increasingly important class of therapeutics. Monoclonal antibodies is the largest and fastest growing class of protein pharmaceuticals [1]. In the discovery and development of these antibodies, antibody fragments such as the antigen binding fragment (Fab), the single-chain variable fragment (scFv), and the single variable domains (V_H_ and V_L_, collectively sdAb) are often employed [2]. The present recombinant antibody discovery platforms, such as ribosome and phage display [3], enable easy screening and selection of antibody fragments against virtually any antigen [4]. Based on the initial screening or selection, a number of candidate antibodies are obtained [3]. Often, these recombinant antibody candidates can be expressed in *E. coli*. However, although the field of recombinant protein expression in *E. coli* is developed and expanded [5,6], the codon usage and folding dynamics of some recombinant antibody clones are incompatible with the bacterial expression machinery [7,8]. In addition, for further evaluation of an antibody fragment it can be necessary to test additional formats, including the Fc-fusion format; such formats are inherently unsuitable for (but not outright incompatible with) prokaryotic expression [7,9].

Modifying a Fab, scFv, or sdAb by fusing them to the Fc-region will produce a bivalent antibody format similar to the canonical antibody [10,11]. The bivalent format increases the apparent affinity due to avidity, provided that multiple epitopes are available.

A further benefit of the Fc-fusion that potentially can be imparted to some antibody fragments is a decrease in their propensity to aggregate [1,12]. At the same time the molecular sizes of the Fc-fused antibodies increase from approximately 12, 25, or 50 kDa (sdAb, scFv, and Fab respectively) to approximately 75, 100, or 150 kDa. An increase of molecular size in this range will greatly increase the serum half-life of a recombinant antibody, by putting it beyond the cut-off for renal clearance. For example native IgG1 of approximately 150 kDa has a serum half-life of around 21 days, whereas the serum half-lives of sdAb and scFv are in the area of 0.05 and 0.1 days respectively [13]. The longer serum half-life of native IgG and of some of the larger recombinant formats is only partly attributed to their molecular size. The longer serum half-life is furthermore a consequence of the interactions of the Fc-region with the neonatal Fc receptor (FcRn). The interaction with FcRn salvages the antibodies from endosomes and returns them to circulation, rather than letting them enter the lysosomal degradation pathway [13,14]. On the other hand, increasing size in general reduces the ability of the antibody fusion to penetrate tissue. The aspects of and need for prolonging the half-life of popular small antibody formats are reviewed in Kontermann 2009 [15].

Besides extending the serum half-life of potential protein therapeutics the Fc-region also confer other useful properties with regard to purification and immunochemistry. In protein purification, the Fc-region allows binding to protein A and protein G, hence supporting effective one step purification by affinity chromatography [16,17]. With respect to the immunochemistry the presence of the Fc-region facilitates detection using many common secondary antibodies [18]. Other obvious tags for detection and purification such as His-tag and C-myc tag are also present in our vector. One should nonetheless also consider protein L for purification of those antibodies holding a kappa light chain. We thus present a vector construct, which allows for versatile strategies of purification and immunochemistry.

The current method of choice for experimental scale expression of full-length antibody, and for formats including the Fc-region, is transient expression in mammalian hosts, such as Chinese hamster ovary cells (CHO) or Human Embryonic Kidney cells (HEK) [19,20]. In such systems, high expression levels can be obtained reaching triple-digit mg/L levels. The drawbacks of mammalian cell expression systems are the need for dedicated labs and equipment, the labour intensive handling, and the economic considerations such as the price of reagents, culture medium, and labware [21].

Expression of recombinant proteins is one of the most central disciplines in molecular biology and medical research, and innovative systems with unique advantages are thus constantly being explored [21]. Among emerging systems is the one based on *L. tarentolae*, a protozoan parasite infecting the gecko *Tarentolae annularis.* The unicellular eukaryote *L. tarentolae* is an extensively studied model for the disease leishmaniasis [22]. Their unique transcriptional and translational machinery has enabled the generation of novel expression systems [23,24] based on the use of exogenous RNA polymerases [25]. Properties like non-laborious handling, post-translational modifications similar to those of mammalian systems, episomal vector maintenance, and effective secretion of recombinant proteins, are some of the strengths of the *L. tarentolae* systems. Recent studies aimed at addressing the importance of the signal peptide cleavage site have shown that expression of scFv antibodies (3.83 mg/L culture medium) can be obtained using this system [26].

In order to validate the system for expression of Fc-fused antibody fragments, we have created a vector allowing expression and secretion of scFvs fused with the Fc-region of rabbit IgG (rFc) using *L. tarentolae* T7-TR (Jena Bioscience). The rFc is a convenient choice when the fusion proteins are to be used in immunochemical analysis of cells (ICC) and tissues (IHC) of human or mice. This is essential to the research performed at laboratories, in which cells and tissues are of human and mice origin, are used. Using the rFc region decreases the interaction, if any, between the endogenous Fc-receptors and the rabbit Fc part. The use of this rFc construct thus reduces the need to block the endogenous Fc receptors of human and mouse tissue [27].

In summary, we here report a system based on *L. tarentolae* T7-TR, for the production of rFc-fused scFv antibodies. The resulting recombinant antibodies are ideal for IHC or ICT on human or rodent material, have a higher apparent affinity for multivalent antigens due to avidity, and are compatible with convenient purification methods. The expression host is characterised by non-laborious handling requirements, eukaryotic post-translational modification of expressed proteins with near-mammalian N-glycan structures, and effective secretion of recombinant protein to the culture medium [28].

## Results and discussion

### Construction of the vector pMJ-LEXSY-rFc for episomal expression-secretion of scFv-rFc

**Figure 1:**
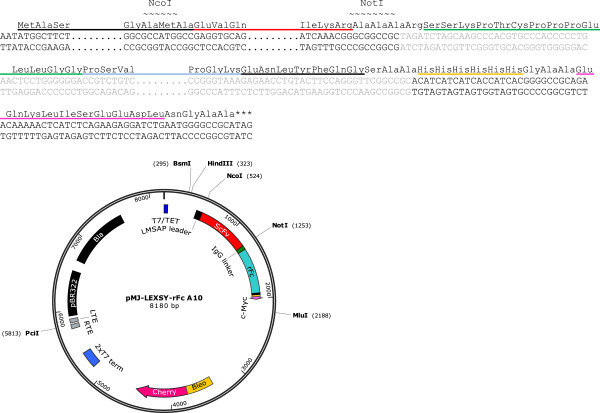
**Description of vector pMJ-LEXSY-rFc.** Top: DNA and protein sequence of cloning site and tag region of pMJ-LEXSY-rFc. Bottom: Vectormap of pMJ-LEXSY-rFc. The T7/Tet region (blue) designates the T7 promoter under control of a Tet operator. LMSAP (black) is the secretory leader-peptide of Secreted Acid Phosphatase 1 found in Leishmania Mexicana. The scFv A10 (red) is inserted via *Nco*I and *Not*I. The Fc-encoding region of rabbit IgG (turquoise) including the IgG linker (green) is placed just downstream of *Not*I followed by a Tev restriction site (black), a His-tag (yellow), and a c-Myc-tag (pink) encoding region. The bleomycin resistance gene is fused with a cherry gene, hence designated BleCherry. This combination allows for selection of recombinants with bleomycin and subsequent screening of the best expressing clones by monitoring the cherry fluorescence. The *Pci*I restriction site is used for linearization and is placed in a telomere region (LTE + RTE), which upon transformation stabilises the linear episome. Finally, the vector holds a tandem T7 transcription terminator (2xT7), a bacterial origin of replication (PBR322 ori), and the Bla ampicillin resistance marker.

The commercial episomal expression system based on *L. tarentolae* T7-TR (Jena Bioscience) is appealing as the target protein can be isolated directly from the culture supernatant, enabling convenient one-step affinity purification. In addition, expression in *L. tarentolae* facilitates post-translational modifications of proteins from higher mammals [28]. However, the vector pLEXSY_IE-blecherry4 does not allow for cloning via *Nco*I/*Not*I, a routinely used restriction enzyme combination for antibody fragments [29], as this will remove the secretory signal peptide and the polyhistidine stretch (His-tag). To render *Nco*I/*Not*I cloning possible we replaced the existing expression cassette with a cassette from a modified version of the pF4SPImsapX1.4sat vector using *Bgl*II and *Mlu*I (own unpublished work). The expression cassette from this vector comprises an *Nco*I/*Not*I cloning site downstream from the signal peptide of *L. mexicana* secreted acid phosphatase 1 (LMSAP1) and a C-terminal His-tag for purification followed by a c-Myc-tag for detection. Replacement of the expression cassette was done without changing the untranslated regions of the parent vector; pLEXSY_IE-blecherry4. The new vector was named pMJ-LEXSY. Moreover, for production of Fc-fusion antibodies, we integrated the rabbit IgG Fc-encoding region (hinge region and CH2-CH3) into the pMJ-LEXSY vector whereby the pMJ-LEXSY-rFc vector was generated (Figure 1). The rFc-encoding region was inserted into the *Not*I site. The insert was prepared using sticky PCR at one end of the insert, resulting in deletion of the plus strand 3′ *Not*I site proximal to the tags upon insertion of the rFc-encoding sequence [30]. Thus the unique *Not*I restriction site was retained, allowing *Nco*I/*Not*I insertion of antibody fragments.

### Expression and purification

**Figure 2:**
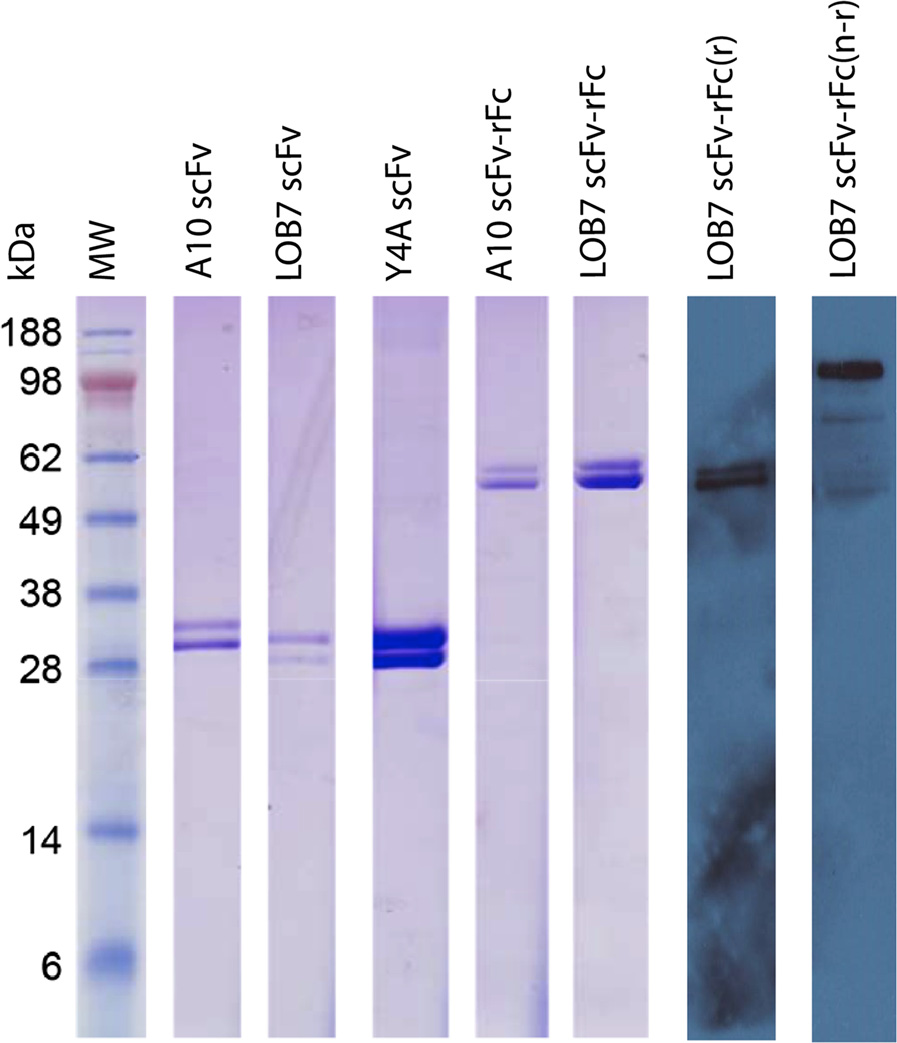
**Antibodies expressed and purified from T7-TR.** Coomasie from the left: Molecular weight marker (SeeBlue plus2; Invitrogen), A10 scFv, LOB7 scFv, Y4A scFv, A10 scFv-rFc, and LOB7 scFv-rFc. Western blot from the left: LOB7-rFc reduced (r), and LOB7-rFc non-reduced (n-r). All antibodies seen in the Coomasie stain were separated on SDS in reducing conditions.

Expressions (80–100 mL in 250 mL flasks) of the two scFv-rFc constructs were carried out for 68-73 hours to explore the level of expression at small-scale. In addition, we explored the capacity of the expression system to produce classical scFvs using the vector pMJ-LEXSY. Three different scFvs, A10, LOB7, and Y4A were expressed together with A10-rFc and LOB7-rFc in *L. tarentolae* T7-TR (Figure 2). The antibody A10 is an scFv derived from the Tomlinson I library [31]. It binds to murine laminin from Engelbreth-Holm-Swarm tumour cells. The antibody LOB7 in an scFv derived from the Tomlinson J library [31]. This antibody binds to human vimentin. The Y4A antibody is an scFv with lambda light chain derived from the YAMO library [32]. This antibody recognises C5a anaphylatoxin. These antibodies were chosen as model antibody fragments, as we had significant experience in expressing them as scFvs in *E. coli*.

Expression and subsequent purification by affinity chromatography established that recombinant antibody fragments could be obtained from all supernatants (Figure 2). Purifications were carried out using Protein A chromatography for the rFc constructs and Ni-NTA Protein Purification for the scFvs. The expression levels observed for the various clones ranged from 0.3 mg/L to 1 mg/L for the scFvs and from 0.6 mg/L to 2.95 mg/L for the rFc-scFvs. Recently, Jäger et al. have shown that yields up to 600 mg/mL of scFv-Fc fusion proteins can be obtained by optimised transient expression in HEK 293 cells [19]. Although the yields in our work is significantly lower, we still consider expression in *L. tarentolae* beneficial due to the few requirements of implementing this system into the laboratory and the non-laborious handling of *L. tarentolae*.

When the purified proteins were analysed by SDS-PAGE two bands were detected. This was also confirmed by the western blot analysis (Figure 2) in which the LOB7-rFc was detected with an HRP conjugated anti-rabbit antibody. Additionally, the western blot analysis could also confirm that the rFc-conjugated antibody assembles into a bivalent structure in non-reducing conditions. The same trend was seen for the A10-rFc antibody (Additional file 1). The apparent heterogeneity of size can be difficult to see for the non-reduced band in the western blot. This is due to the fact that the size difference of the two bands is relatively smaller to the total mass of the assembled bivalent antibody, hence giving a lower separation. This apparent heterogeneity of size was seen for all constructs; scFvs (28 kDa) and scfv-rFcs (55 kDa) (Figure 2). To investigate this issue we utilised that the pMJ-LEXSY-rFc vector (Figure 1) enables the removal of the His-tag and the c-Myc-tag by TEV protease digestion. By removing the c-terminal tags with TEV Protease it was possible to assess whether the source of size heterogeneity was placed in the tag-region. SDS-PAGE analysis of LOB7-rFc digested with TEV Protease showed that one band appeared on the gel as opposed to two bands from non-digested sample, confirming that the apparent heterogeneity of size was due to unpredicted modifications of the tag-region (Additional file 2: Figure S1). Furthermore, western blot analysis and mass spectrometry on the Y4A-scFv strongly suggest that the heterogeneity of size emerges from a truncation of the c-Myc tag in the lower molecular-weight protein (Additional file 2: Figure S2-S4). The truncation of the c-Myc tag has however no obvious influence on the intended application of the scFv-rFcs in IHC and ICC using secondary antibodies targeting the rFc-region.

### Functionality

**Figure 3:**
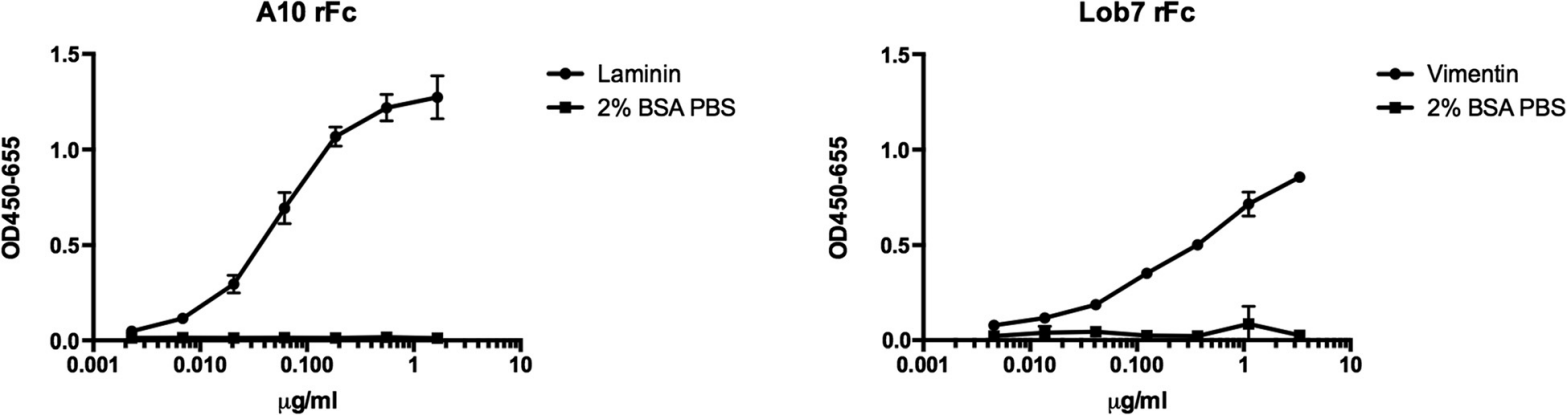
**ELISA; specific binding of rFc fusion scFvs against their cognate antigens.** To test if the scFv-rFc antibodies bind their cognate antigens ELISA plates were coated with laminin (A10 scFv-rFc) and vimentin (LOB7 scFv-rFc). For both antibody constructs specific binding is seen.

To assess the binding activity of the scFv-rFcs, ELISAs were performed (Figure 3) with A10-rFc and LOB7-rFc against their respective antigens (mouse laminin and human vimentin). The antigens were coated on ELISA plates, targeted by the scFv-rFcs, and detected using polyclonal swine anti-rabbit immunoglobulin HRP.

**Figure 4:**
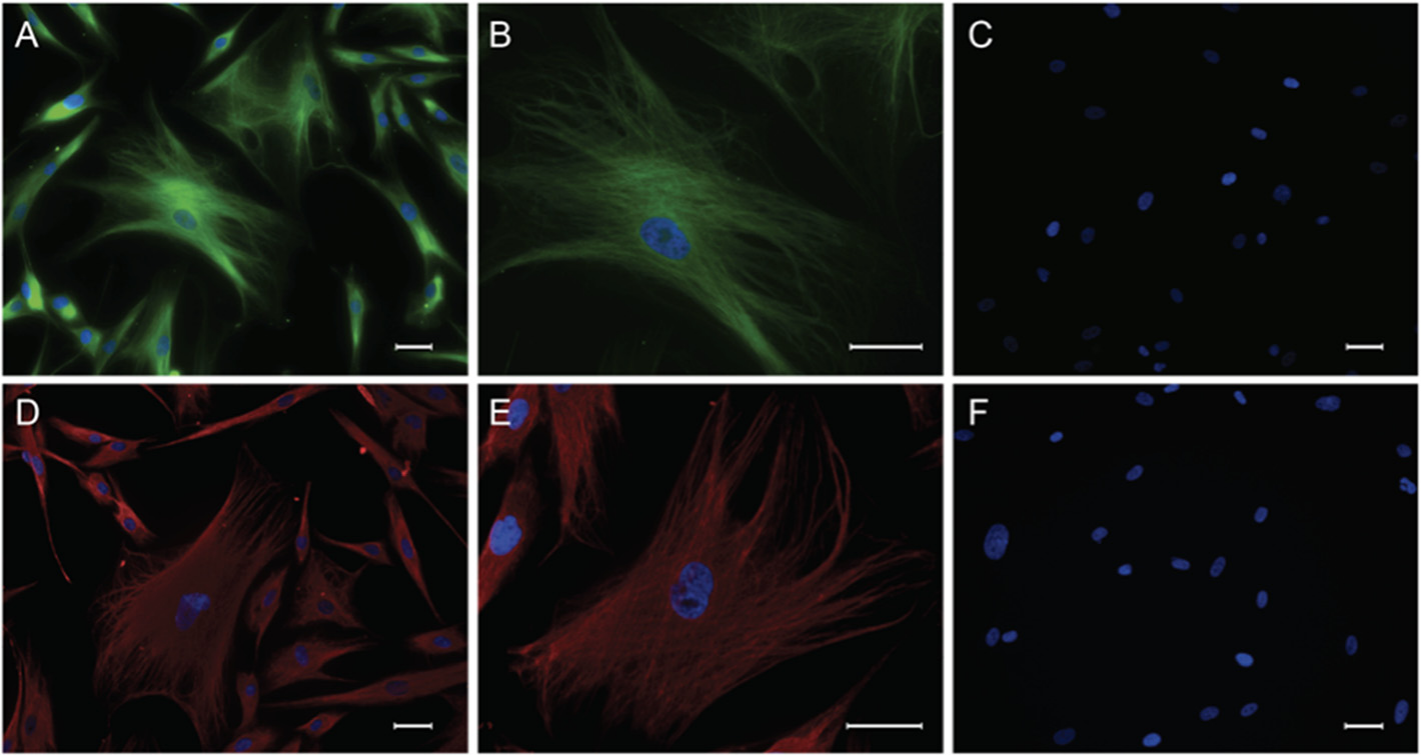
**Using LOB7-rFc and V9 antibody for Immunocytochemistry.** The cell line ASF-2 were stained with LOB7 scFv-rFc **(A, B)** and V9 **(D, E)** and detected with Goat-anti-Rabbit Alexa Fluor 488 (green) and Goat-anti-Mouse Alexa Fluor 546 (red) respectively. Negative controls **(C, F)** were done leaving out primary antibodies. The cell nuclei were stained with DAPI (blue). Both LOB7 and V9 detect vimentin, a cytoplasmic intermediate filament. Both give excellent staining. (Scale bar, 20 μm).

The rFc-constructs show specific binding to their cognate antigens. Furthermore, the scFv-rFcs were detectable using HRP-conjugated anti-rabbit immunoglobulin. The detection with the secondary antibody shows that the rFc-regions were functionally intact, in line with the fact that the antibodies can be purified using protein A affinity chromatography [33]. On this basis we conclude that our system is suitable for the production of rFc-fused antibody fragments with uncompromised folding. To further establish the capacity of this system for production of antibodies intended for immunochemistry, LOB7-rFc was used for immunocytochemical staining of fixed and permeabilised human adult skin fibroblasts (ASF-2) [34]. A commercial mouse monoclonal antibody (V9) directed against vimentin was included as a benchmark for detection fidelity (Figure 4).

Both antibodies target vimentin, a cytoplasmic intermediate filament. As can be seen, LOB7-rFc and commercial V9 antibody produces similar labelling patterns when binding to vimentin.

## Conclusions

Antibody fragments fused to the Fc-region of IgG have previously been produced in several organisms [35-39], but to our knowledge this is the first time such constructs have been expressed in *L. tarentolae*. The *L.tarentolae* system offers a system capable of making N-glycosylations and O-glycosylations. Breitling et al. reported the production of biological active biantennary homogenously N-glycosylated hEPO in L. tarentolae [23]. The N-glycosylations performed in L. tarentolae are of higher mammal resemblance than those performed in yeast and insect cells. Moreover, studies by Klatt et al. [40] have shown that ssAlpha expressed in L. tarentolae are O-glycosylated at the same sites as in mammal cells and furthermore displayed an increased resistance to degradation compared to ssAplpha expressed in *E. coli*[40].

At the same time *L. tarentolae* expression cultures can be handled under standard laboratory conditions, similar to the conditions used for propagation of *E. coli* cultures. For the production of antibody fragments resulting from high throughput screening, the system we describe here presents substantial benefits: Firstly, the system can provide a rescue avenue for antibody fragments that express poorly in *E. coli*. Secondly, the system can provide recombinant antibodies in a bivalent format incorporating a native Fc-region; a format ideally suited for use in immunostaining of cells and tissues.

## Methods and material

### Vector construction

All restriction enzymes, polymerases, and chemicals used for cloning and PCR were purchased from Fermentas (Thermo Scientific Molecular Biology). Oligonucleotides used are listed in Table 1, and were purchased from Sigma Aldrich. To make the pLEXSY_IE-blecherry4 applicable for *Nco*I/*Not*I cloning of scFv antibodies for secretion an expression cassette from the vector pF4NAF was cloned into pLEXSY_IE-blecherry4 via *Bgl*I and *Mlu*I. The pF4NAF expression cassette had been constructed previously using components of two vectors; pIT2 [31] and pF4SPImsapX1.4sat (Jena Bioscience; Cat. No. EGE-211). In this previous work, the vector pF4SPImsapX1.4sat (an early version of pLEXSY-sat2, Jena Bioscience; Cat. No. EGE-234) was modified by deleting the *Nco*I with primer Z and primer X, by use of PCR amplification of the region between *Bgl*II and *Not*I. The PCR product, now deleted for *Nco*I, was subsequently digested with *Bgl*II and *Not*I and re-inserted into the pF4SPImsapX1.4sat. Next, the expression cassette from the vector pIT2 was PCR amplified using primer A and primer B, followed by a digestion of the PCR product with *Kas*I (5′ digestion) and *Bsp120*I (3′ digestion). *Bsp120*I produces overhangs compatible with *Not*I. The PCR amplified expression cassette of pIT2 was then inserted into pF4SPImsapX1.4sat via *Kas*I and *Not*I hereby creating the pF4NAF cassette. In the ligation of the *Bsp120*I overhang to the *Not*I overhang the recognition sequence for both was destroyed. This cassette was inserted into pLEXSY_IE-blecherry4 via *Bgl*I and *Mlu*I, resulting in pMJ-LEXSY. The ligated DNA was used to transform electrocompetent XL1-Blue, and the cells were plated on TYE agar-plates containing 100 μg/mL ampicillin (Sigma Aldrich). The plates were incubated overnight at 30°C and colonies were picked for sequence analysis. To construct the rFc-fusion vector pMJ-LEXSY-rFc, the Fc encoding region of rabbit IgG (hinge region and CH2-CH3) was amplified by two rounds of PCR amplification using the vector pFUSE-rIgG-Fc2 as template [18]. The two PCR amplifications were performed applying primer pairs c/d and c/e. respectively. The rFc-encoding region was prepared for cloning using sticky end PCR as earlier described [30] and inserted into the *Not*I site of pMJ-LEXSY. The Ligations were electroporated into XL1-Blue and plated on agar-plates holding 100 μg/mL ampicillin. Colony PCR was used to identify positive clones and correct orientation of DNA inserts were verified by sequencing (Eurofins MWG).

**Table 1:** Primers used in this study


Primer z	5′ GCAGCC*AGATCT*AATATGGCTTCTAGGCTCGTCCG 3′
Primer x	5′ AGGAGG*GCGGCCGC*TTA ′3
Primer A	5′ CGCGTG*GGCGCC*ATGGCCGAGGTGCAG 3′
Primer B	5′ CTGCAA*GGGCCC*CTATGCGGCCCCATTCAGATC 3′
Sticky.frw	5′ TACCTT*GCGGCCGC*TAGATCTAGCAAG 3′
Sticky.short.rev	5′ GAACCCTGGAAGTACAGGTTCTCTTTACCCGGAGAGCGGGAGATG 3′
Sticky.long.rev	5′ GGCCGAACCCTGGAAGTACAGGTTCTCTTTACCCGGAGAGCGGGAGATG 3′

### Cultivation and transformation

#### XL1-Blue

Before transformation, XL1-Blue cells were made electrocompetent roughly as described for *Pseudomonas Putida* in [41], but without adding sucrose to the storage medium. Batches of competent *E. coli* were frozen in liquid nitrogen and stored at -80°C. Electroporation was carried out in 2 mm pre-chilled cuvettes holding 20-30 ng of vector and 50 μL cells. The cells were pulsed at 2500 V using an Electroporator 2510 (Eppendorf) and plated on TYE agar-plates with 100 μg/mL ampicillin. For plasmid propagation, XL1-Blue were grown at 30°C and 200 rpm in baffled Erlenmeyer flasks containing PDM medium [42] with 100 μg/mL ampicillin.

#### Leishmania tarentolae T7-TR

Manipulation of T7-TR was performed in accordance with Jena Bioscience protocols (Cat. No. EGE-1420blecherry). In brief, T7-TR were cultivated and maintained in tissue culture flasks (TC25) as static suspensions at 26°C. The flasks were placed upright, in the dark, and with the ventilating cap in the open position. Non-transformed *Leishmania tarentolae* T7-TR were grown in 10–15 mL LEXSY BHI medium per flask. The cells were passaged twice weekly in ratios of 1:50 or 1:20 on Mondays and Fridays respectively. For transformation, 10 μg of linearized DNA in 50 μL of water was incubated with 350 μL densely suspended *Leishmania tarentolae* T7-TR (OD_600_ > 2). Electroporation was carried out in pre-chilled 2 mm cuvettes. Cells were pulsed in a GENEPULSER Xcell (BIORAD) at 450 V and 450 μF, obtaining a pulse time in ranges of 5-6.5 ms. The cells were kept on ice for exactly 10 minutes after electroporation. Immediately hereafter the cells were transferred to a T25 flask and grown for 20 hours in 10 mL of non-selective LEXSY BHI medium. Cells were subsequently gently dispensed onto selective LEXSY BHI agar plates and grown for 10 days at 26°C in the dark. The plates for clonal selection contained 100 μg/mL Zeocin (Invitrogen). All colonies visible after 10 days were picked from the plates, and each clone was then cultivated in selective LEXSY BHI medium for one day in a single well of a 24-well plate. This cultivation was performed in 1 mL selective LEXSY BHI medium with 100 μg/mL zeocin (Invitrogen). The cell density and condition was assessed in the microscope and clones with low growth were cultivated for further 1–2 days before they were transferred to a larger volume. Clones exhibiting acceptable motility, cell shape, and growth were transferred to 5 mL selective LEXSY BHI medium and grown to OD 1.4. Assessment of expression levels for each clone was conducted in 96-well plates by inducing the expression with 100 μg/mL tetracycline. The cherry fluorescence of each clone was measured at 584 nm excitation/612 nm emission in a POLARstar OPTIMA fluorimeter (BMG Labtech).

### Expression and purification

Clones displaying the highest level of cherry fluorescence were chosen for the further work. Expression was carried out in 100 mL LEXSY BHI medium containing 100 μg/mL tetracycline (Sigma Aldrich) and 100 μg/mL Zeocin (Invitrogen). Transformed *L. tarentolae* T7-TR (OD_600_ 1.4-2) were innoculated 1:10 into 100 mL cultures. The culturing was performed in the dark at 26°C for 72 hours in 250 mL baffled Erlenmeyer flasks with agitation (120 rpm). The cultures were centrifuged at 2700 × *g* for 30 min to precipitate cells and the proteins present in the supernatant precipitated by using 30% m/v ammonium sulphate. The precipitated proteins were then pelleted at 5250 × *g* for 45 min and re-suspended in 0.5 × PBS. Finally, affinity purification using a Protein A HP spinTrap column (GE healthcare) was used for recovery of the scFv-rFc antibodies. The scFv antibodies were purified using a Maxwell 16 instrument in combination with a Maxwell 16 Polyhistidine Protein Purification Kit (Promega). Protein concentrations were estimated by absorption at 280 nm using a NanoDrop 1000 instrument (Thermo Scientific) and applying the protein specific molecular weights and molar extinction coefficients. A repeated and up-scaled expression of LOB7-rFc was performed as described above, but this time as 5 times 80 mL cultures incubated for 72 hours in 250 mL baffled Erlenmeyer flasks. Supernatants from all cultures were pooled before the proteins were precipitated. The ammonium sulphate precipitated proteins were then resuspended in 20 mM sodium phosphate and purified by using a 1 mL HiTrap Protein A HP column. Protein A purification was carried out as outlined by the manufacturer (GE Healthcare).

### Functionality

#### ELISA

Maxisorp 96-well flat bottom plates (Nunc) were coated with 30 μL of the relevant antigens at 20 μg/mL (in 2% BSA-PBS) for both vimentin and laminin. The protein was adsorbed to the plates during storage at 4°C overnight. Each well was washed with three times 200 μL PBS using a multi-channel pipette, before the plates were blocked with 300 μL 2% BSA in PBS (BSA-PBS) for 1 hour at room temperature under gentle agitation. The washing step was then repeated, followed by the addition of serial dilutions of scFv-rFcs in 100 μL 2% BSA-PBS. The antibodies were incubated in the antigen-coated wells for 1.5 hour at room temperature, and subsequently the plates were washed three times with PBS, each time for 5 min. Detection of bound scFv-rFcs was performed by incubating the plates with polyclonal swine anti-Rabbit Immunoglobulins (Dako) in a 1:2000 dilution in 2% BSA-PBS for 1 hour. Finally, the plates were washed three times with PBS, each time for 5 minutes. The amount of bound antibody was visualised by the addition of 80 uL TMB single solution (Sigma Aldrich), and the colour reaction was terminated after 7 minutes by the addition of 50 μL 1 M H_2_SO_4_ to each well. Absorbance was measured at 450 nm and corrected for at 655 nm. The absorbance was conducted in a microplate reader; Model 550 (Bio Rad).

#### Eukaryotic cell handling and Immunocytochemistry

ASF-2 cells were grown in DMEM (Lonza) with 10% fetal bovine serum (Thermo Scientific), 100 U/mL penicillin and streptomycin (Lonza) at 37°C, 5% CO_2_ and 95% humidity. ASF-2 cells of passage 10 were detached from a tissue culture flask by trypsination with Trypsin EDTA (Lonza) and spun down at 600 g for 6 minutes. Cells were then resuspended in growth medium and 15.000 cells/well were seeded out in an ibiTreat μ-Slide VI 0.4 (Ibidi) and grown overnight. The next day the cells were rinsed with PBS, fixed in 4% PFA for 15 min., permeabilised with 0.025% Triton X100 (Sigma-Aldrich) for 10 min. and blocked with 2% BSA in PBS (BSA-PBS) for 1 hour at room temperature. The cells were incubated with 5 μg of purified LOB7 in 2% BSA-PBS or 100 μl of a 1:100 dilution of V9 antibody in 2% BSA-PBS (Sigma Aldrich) pr. well for 1 hour at room temperature. Visualisation of LOB7 was accomplished by incubation with a 1:100 dilution of Goat-anti-Rabbit Alexa Fluor 488 (Invitrogen, USA). V9 was visualised by a 1:100 dilution of Goat-anti-Mouse Alexa Fluor 546 (Invitrogen). Cell nuclei were stained with Vectashield Mounting Medium with DAPI (Vector Labs). Fluorescent images were obtained with a Leica DMI3000 B inverted microscope (Leica Microsystems).

## Competing interests

The authors declare that they have no competing interests.

## Authors’ contributions

MLJ, NAF and PK contributed to the design of the study and MLJ and NAF performed the majority of the experiments. JJ performed immunocytochemistry, PM participated in the manipulation of L. Tarentolae. SVP performed the mass spectrometry. All authors participated in writing and critical review of the manuscript.

## Supplementary Material

Additional file 1 SDS PAGE showing reduced and non-reduced rFc constructs. Click here for file

Additional file 2: Figure S1 SDS PAGE analysis of TEV Protease digested LOB7-rFc. To assess if the heterogeneity correlated to modifications of the C-terminal tag region, we digested LOB7-rFc with TEV Protease. (1) Non-digested LOB7-rFc showing two bands (2) LOB7-rFc digested with TEV Protease showing one band. Therefore, the size heterogeneity resides in the C-terminal tag-region. **Figure S2 - Western blot analysis of Y4A-scFv.** (A) Western blot analysis of 0.018 μg and 0.09 μg Y4A-scFv using an anti-His antibody. Two bands appeared after exposure for 1 min and 10 sec, respectively. (B) Western blot analysis on 0.45 μg Y4A-scFv using an anti c-Myc antibody. The films were exposed 1 min and 10 sec, displaying only one band. (C) Coomasie stain of the Y4A-scFv. **Figure S3 - Verification of degradation of c-Myc-tag by mass spectrometry (MS).** The bands detected by SDS-PAGE were subjected to in-gel digestion using Lys-C and the peptides were subsequently analysed by MALDI-TOF mass spectrometry. Ions in the range m/z 1500–2500 are shown. An ion of m/z 2221.28 was detected in the upper band whereas an ion of m/z 1693.91 in the lower band. The mass difference (~500 Da.) correlates with the mass difference observed by SDS-PAGE. **Figure S4 - MSMS analysis of heterogeneous antibody.** To evaluate the identity of the ions detected, we subjected them to MSMS analysis. (A) The analysis of the ion detected in the upper band produced fragment ions corresponding to the peptide represented by Leu239-Lys258 encompassing the His-tag and three amino acid residues of the c-myc tag. The C-terminal Lys258 indicates that this peptide is generated by Lys-C cleavage. (B) The ion of m/z 1693.91 was found to represent Leu239-Gly253. It is thus likely that the C-terminal Gly253 represents the C-terminus of the mature protein excised from the gel (lower band). Click here for file
