# Supplementary figures and images for: Expression of single-chain variable fragments fused with the Fc-region of rabbit IgG in Leishmania tarentolae
Source: Microb Cell Fact. 2014 Jan 15;13:9. doi: 10.1186/1475-2859-13-9 (PMC3917567; doi:10.1186/1475-2859-13-9)

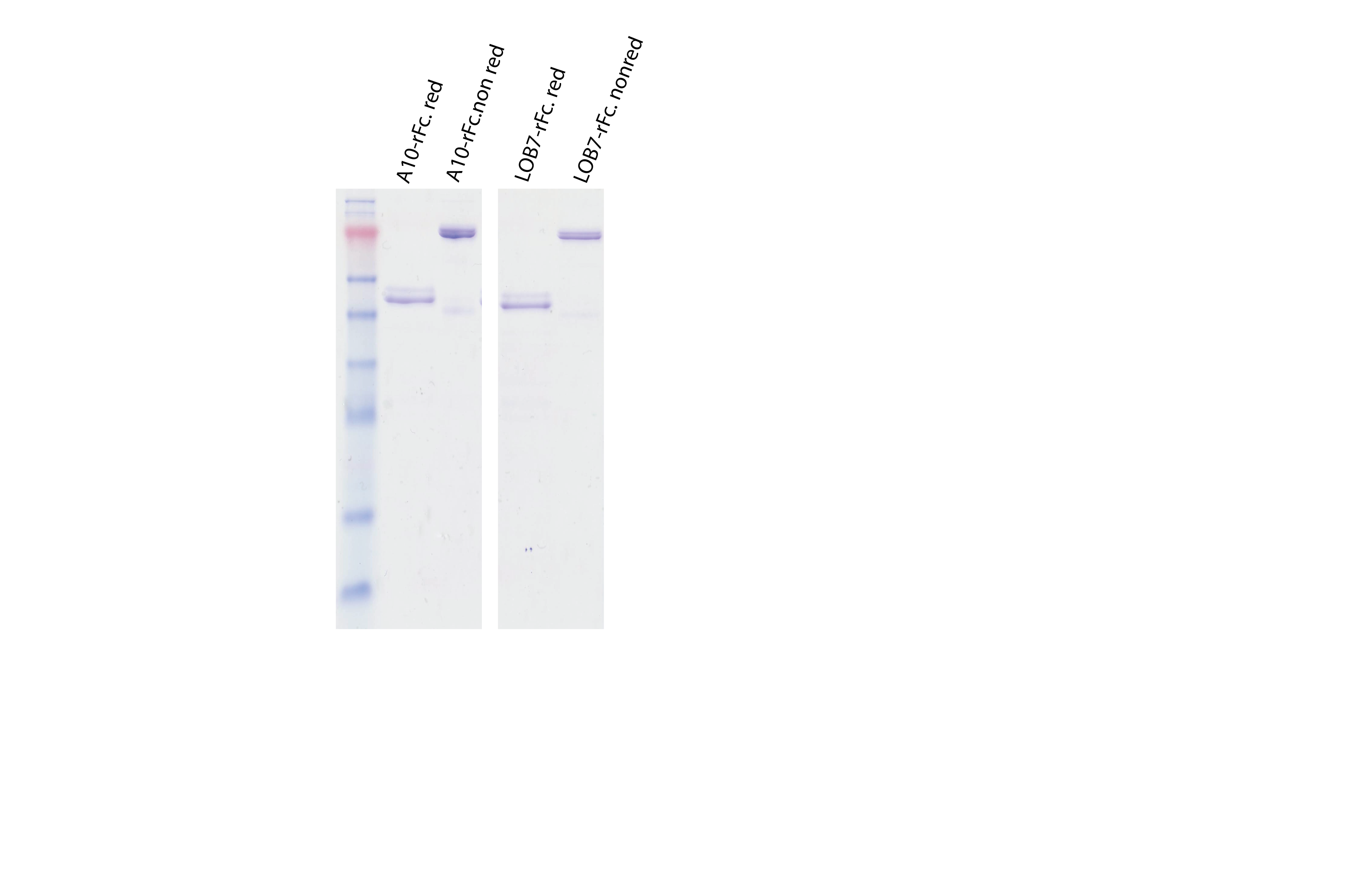

Supplement: Additional file 1 — SDS PAGE showing reduced and non-reduced rFc constructs. [file 1475-2859-13-9-S1.png]

Figure S1

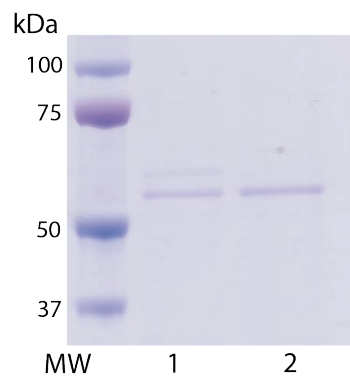

Figure S2

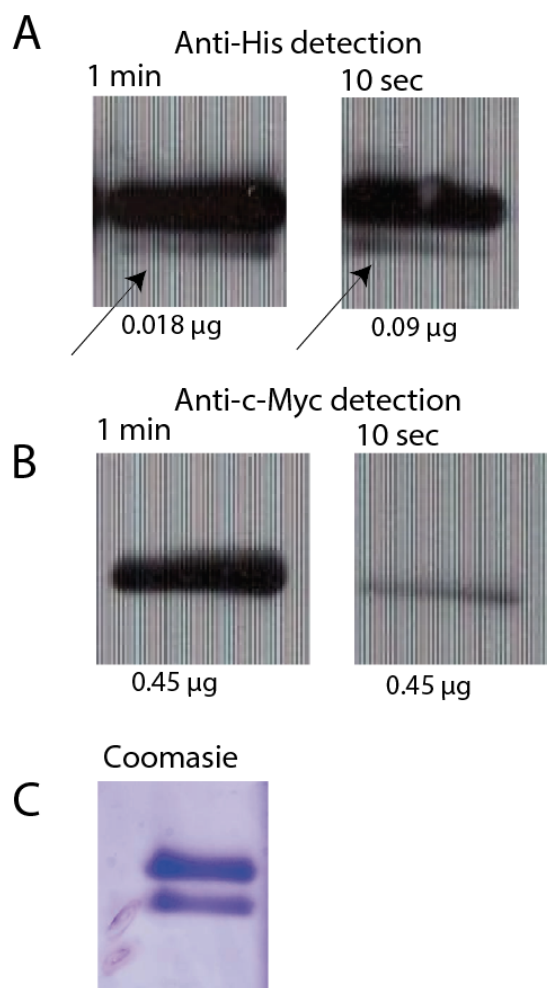

Figure S3

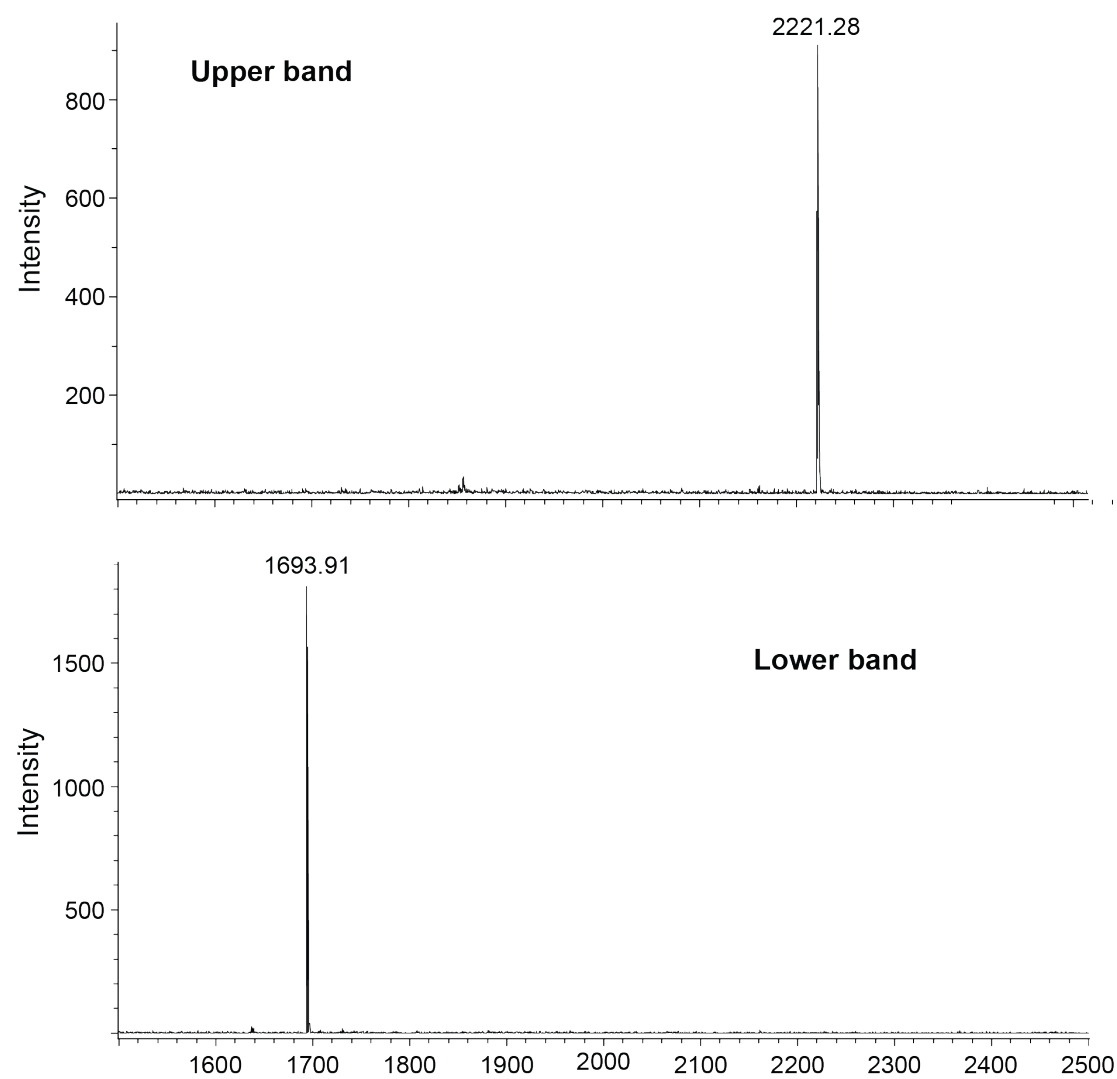

Figure S4

**A**

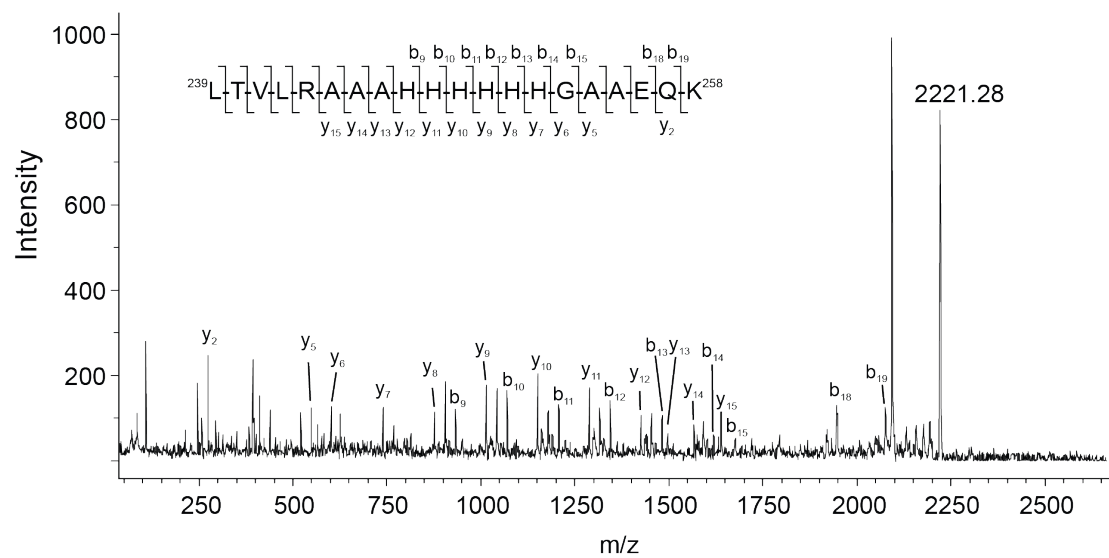

**B**

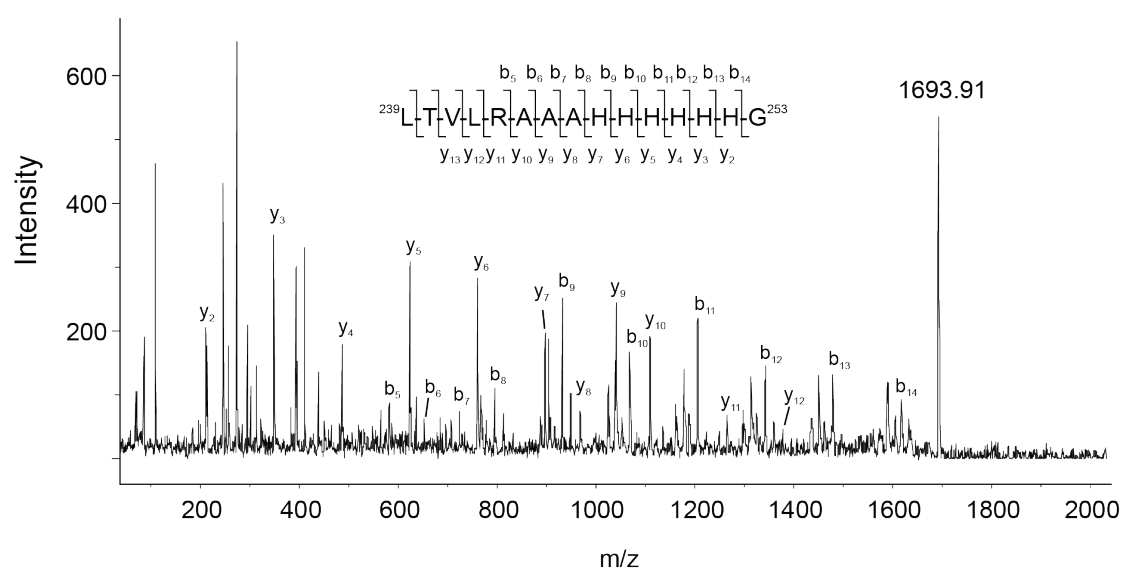

Supplement: Additional file 2: Figure S1 — SDS PAGE analysis of TEV Protease digested LOB7-rFc. To assess if the heterogeneity correlated to modifications of the C-terminal tag region, we digested LOB7-rFc with TEV Protease. (1) Non-digested LOB7-rFc showing two bands (2) LOB7-rFc digested with TEV Protease showing one band. Therefore, the size heterogeneity resides in the C-terminal tag-region. Figure S2 - Western blot analysis of Y4A-scFv. (A) Western blot analysis of 0.018 μg and 0.09 μg Y4A-scFv using an anti-His antibody. Two bands appeared after exposure for 1 min and 10 sec, respectively. (B) Western blot analysis on 0.45 μg Y4A-scFv using an anti c-Myc antibody. The films were exposed 1 min and 10 sec, displaying only one band. (C) Coomasie stain of the Y4A-scFv. Figure S3 - Verification of degradation of c-Myc-tag by mass spectrometry (MS). The bands detected by SDS-PAGE were subjected to in-gel digestion using Lys-C and the peptides were subsequently analysed by MALDI-TOF mass spectrometry. Ions in the range m/z 1500–2500 are shown. An ion of m/z 2221.28 was detected in the upper band whereas an ion of m/z 1693.91 in the lower band. The mass difference (~500 Da.) correlates with the mass difference observed by SDS-PAGE. Figure S4 - MSMS analysis of heterogeneous antibody. To evaluate the identity of the ions detected, we subjected them to MSMS analysis. (A) The analysis of the ion detected in the upper band produced fragment ions corresponding to the peptide represented by Leu239-Lys258 encompassing the His-tag and three amino acid residues of the c-myc tag. The C-terminal Lys258 indicates that this peptide is generated by Lys-C cleavage. (B) The ion of m/z 1693.91 was found to represent Leu239-Gly253. It is thus likely that the C-terminal Gly253 represents the C-terminus of the mature protein excised from the gel (lower band). [file 1475-2859-13-9-S2.pdf]
